# Microglia and Microglia-Like Cell Differentiated from DC Inhibit CD4 T Cell Proliferation

**DOI:** 10.1371/journal.pone.0007869

**Published:** 2009-11-17

**Authors:** Bo Bai, Wengang Song, Yewei Ji, Xi Liu, Lei Tian, Chao Wang, Dongwei Chen, Xiaoning Zhang, Minghui Zhang

**Affiliations:** 1 Department of Neurobiology, Taishan Medical College, Taian, Shandong Province, People's Republic of China; 2 Institute of Immunology, School of Medicine, Tsinghua University, Beijing, People's Republic of China; Ohio State University, United States of America

## Abstract

The central nervous system (CNS) is generally regarded as a site of immune privilege, whether the antigen presenting cells (APCs) are involved in the immune homeostasis of the CNS is largely unknown. Microglia and DCs are major APCs in physiological and pathological conditions, respectively. In this work, primary microglia and microglia-like cells obtained by co-culturing mature dendritic cells with CNS endothelial cells in vitro were functional evaluated. We found that microglia not only cannot prime CD4 T cells but also inhibit mature DCs (maDCs) initiated CD4 T cells proliferation. More importantly, endothelia from the CNS can differentiate maDCs into microglia-like cells (MLCs), which possess similar phenotype and immune inhibitory function as microglia. Soluble factors including NO lie behind the suppression of CD4 T cell proliferation induced by both microglia and MLCs. All the data indicate that under physiological conditions, microglia play important roles in maintaining immune homeostasis of the CNS, whereas in a pathological situation, the infiltrated DCs can be educated by the local microenvironment and differentiate into MLCs with inhibitory function.

## Introduction

Microglia are resident macrophages located in the central nervous system (CNS) parenchyma, the number is about 5–15% of the cells in the CNS[1], [2]. Because of the low expression of MHC class II, B7 and CD40, the essential molecules necessary for antigen presentation, microglia seem to be potential APCs that can mount the immune response in the CNS[3]. But there are still no evidences to demonstrate that microglia can present antigens to T cells and initiate the immune response in the CNS. The immunological role of microglia in physiological or pathological immune response in the CNS is still a puzzle.

There are few mature DCs (maDC) and lymphocytes in non-inflammatory CNS due to the function of brain blood barrier (BBB), so the CNS is generally regarded as a site of immune privilege[4], [5]. But under pathological conditions, the BBB is broken down and large numbers of immunocytes including monocytes and dendritic cells (DCs) could be recruited to the CNS. In experimental autoimmune encephalomyelitis (EAE), there is a considerable increase of DCs in the CNS during the early stage[6], [7]. But the function of DCs in the CNS of EAE model is contradictory. Some researchers found that DCs isolated from the CNS of EAE model can present peptides to T cells[8], whereas others reported that DCs with the same origin can not[9], [10]. Our previous studies showed that immune microenvironment has great effects on the functions of DCs, so we speculate that the microenvironment of the CNS may lie behind the contradiction of the DCs function in the CNS of EAE model.

Increasing data showed that some antigen presenting cell subtypes, including regulatory dendritic cells and suppressive macrophages with inhibitory function, are important in terminating immune responses for maintaining immune homeostasis[11]. As the protagonist of APC in the CNS, whether microglia will be involved in the local immune homeostasis should be illustrated and the influence of the CNS microenvironment on the function of the DCs recruited to the CNS should be addressed.

In this study, we isolated the microglia from the brain of perfused mouse and tested the antigen presenting ability using OVA-specific-TCR transgenic CD4 T cells as responser. In addition, the CNS endothelia were isolated and cultured to mimic the microenvironment of the CNS in vitro, the effects of the CNS endothelia on the infiltrated DCs and the effects of the educated DCs on T cell activation and proliferation were explored. Our work suggests that microglia and the CNS-endothelia-educated DCs can prevent the proliferation of CD4 T cells and indicates the APCs in the CNS may play an important role in keeping immune homeostasis of the CNS by inhibiting T cell proliferation.

## Results

### Microglia Inhibit maDC-Initiated Proliferation of CD4 T Cells

**Figure 1:**
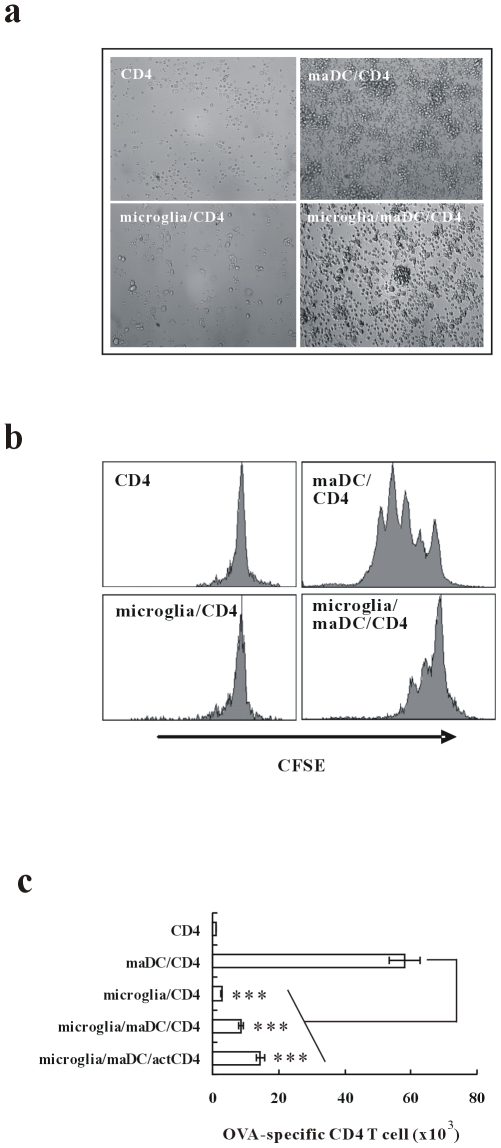
Microglia inhibit maDC-initiated proliferation of CD4 T cells. CD4 T cells from DO11.10×C57BL/6 F1 hybrid mice were cocultured with maDCs or/and microglia for 5 days. (a)The photos were taken in bright field (objective ×20). (b) The proliferation of CFSE-labeled CD4 T cells was detected by FACS after 3 days coculture. (c) The live CD4 T cells in the coculture system were counted by FACS. Data are presented as mean±s.d. of triplicate wells and representative of three independent experiments.^ ***^ *P*<0.01.

We used TCR transgenic CD4^+^ T cells specific for OVA_323−339_ to test the APC function of microglia. As shown in Figure 1a, OVA_323−339_-pulsed maDCs could stimulate T cells to proliferate dramatically but OVA_323−339_-pulsed microglia did not. Moreover, microglia could significantly inhibit maDCs-initiated T cell proliferation when microglia were added into maDC/T cells coculture system simultaneously. Similar result was obtained using CFSE-labeled CD4 T cells (Figure 1b). Furthermore, we found that microglia could inhibit the proliferation of activated T cells which has been cocultured with maDCs for 24 hours (Figure 1c). Putting the data together, microglia not only failed to initiate the proliferation of naïve T cells but also could inhibit maDC-initiated proliferation of naïve or activated T cells.

### The Characteristics of the Endothelial Cells from the CNS

**Figure 2:**
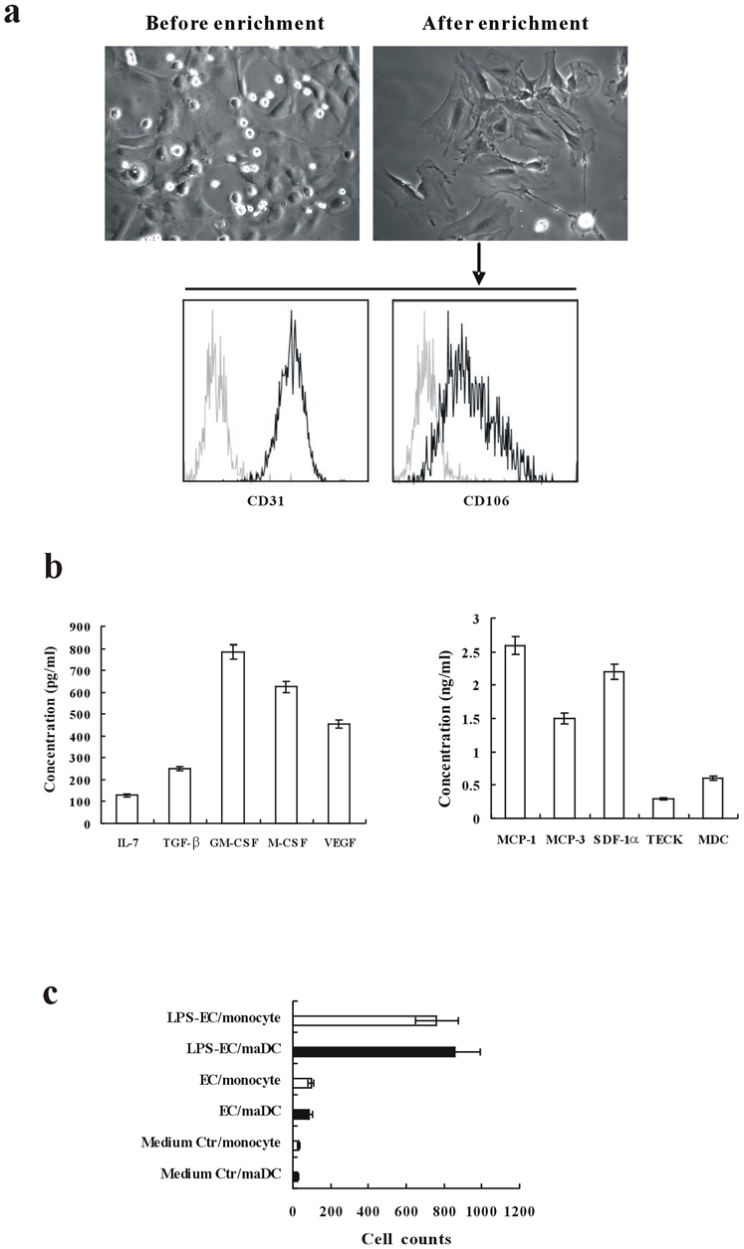
The characteristics of the endothelial cells from the CNS. (a) The CNS endothelial stroma cells before and after CD11b^−^ CD31^+^ selection were observed under the microscopy. The purity of CD11b^−^ CD31^+^ cells was tested by expression of CD31 and CD106. (b, c) The secretion of cytokines including IL-7, TGF-β, GM-CSF, M-CSF and VEGF(b), and chemokines including MCP-1, MCP-3, SDF-1α, TECK and MDC(c) was detected. (d)The chemotactic ability to monocytes or DCs of the supernatant of LPS-treated EC was assayed.

To obtain stromal cells mimicking the microenvironment of the CNS, newborn C57BL/6 mice brain was scissored into pieces and attached to 24-well plate. After 2 weeks, the attached cells were digested and CD11b^−^ CD31^+^ cells were isolated and used as the CNS endothelial stroma cells. Pictures were taken to show the morphology of the endothelial cells before and after enrichment. The purity of CD11b^−^ CD31^+^ cells was tested by CD31 and CD106 using FACS (Figure 2a). Moreover, the secretion of cytokines, including IL-7, TGF-β, GM-CSF, M-CSF and VEGF (Figure 2b), and chemokines including MCP-1, MCP-3, SDF-1α, TECK and MDC (Figure 2c), were detected. The high concentration of MCP-1 indicates that the endothelial cells might chemoattract macrophages or DCs. The chemotactic assay confirmed our hypothesis and the supernatant of LPS-treated EC has a more potent ability to chemoattract monocytes or DCs (Figure 2d).

### Endothelial Cells Induce maDCs to Differentiate into MLCs

**Figure 3:**
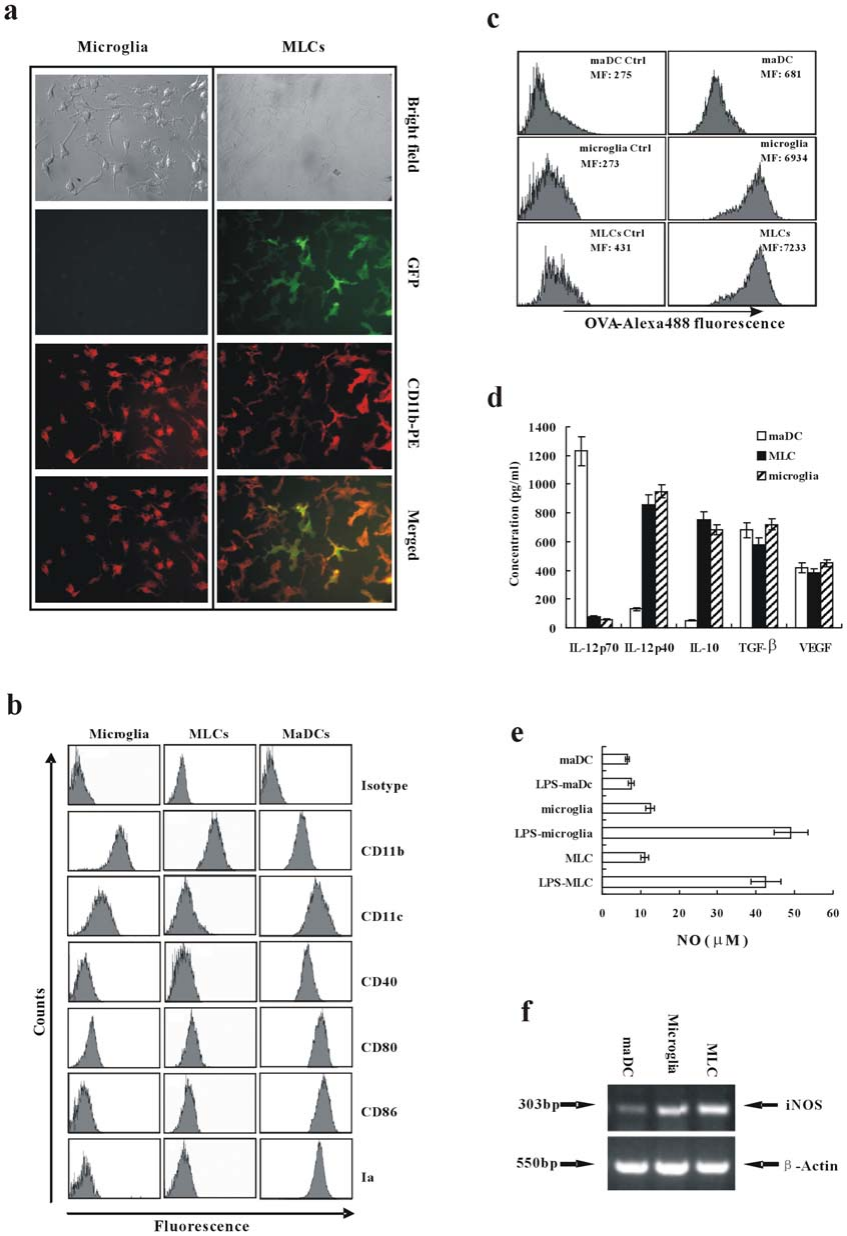
Endothelia induce maDCs to differentiate into MLCs. (a) GFP^+^ maDCs cultured on monolayers of CNS endothelia for 14 days and were labeled with PE-conjugated anti-CD11b antibody. Fluorescence photos were taken with a Leica fluorescent microscope (objective 40×). (b) Comparison of phenotype among microglia, MLCs and maDCs. (c) Comparison of phagocytic ability among microglia, MLCs and maDCs. Numbers in histograms indicate the geometric mean fluorescence. (d) The secretion of IL-12p70, IL-12p40, IL-10, TGF-β1 and VEGF by maDC, microglia and MLCs stimulated by 10 ng/ml LPS for 24 h was detected. (e) The production of NO by maDC, microglia and MLCs stimulated by 10 ng/ml LPS for 24 h was detected. (f) The expression of iNOS in maDC, microglia and MLCs was detected by RT-PCR.

To find out the effect of the CNS microenvironment on the infiltrated DCs, bone marrow maDCs derived from EGFP transgenic mouse were seeded on the endothelia stroma derived from the CNS. The maDCs attached to the endothelia and along with the continuous culture, proliferated on endothelia. After 14 days coculture, maDCs differentiated into cells with typical shape of microglia (for the ensuing results, these differentiated cells were nominated as microglia like cells, MLCs) (Figure 3a). The phenotype of the differentiated EGFP^+^ cells (MLCs) was analyzed. As shown in Figure 3b, these cells express relatively high level of CD11b, low CD11c, MHC class II and costimulatory molecules, such as CD80, CD86 and CD40 compared with mature DCs. Notably, the phenotype of the differentiated cells (MLCs) was quite similar to that of microglia and different from that of maDCs. The high expression of CD11b, a crucial marker of microglia, on the differentiated cells (MLCs) was confirmed under the microscope (Figure.3a). Furthermore, the phagocytic capability assay showed that the differentiated cells (MLCs) displayed notable phagocytic capability, similar with microglia, while maDCs showed poor phagocytic capability (Figure 3c). In cytokine secretion, the results showed that the LPS-treated differentiated cells (MLCs) could produce similar high levels of IL-12p40 as microglia, different from the high secretion of IL-12p70 from mature DC (Figure 3d). Since microglia has inhibitory function, we detected the secretion of IL-10, TGF-β1, VEGF and NO which have been reported to have potential inhibitory effect. The results showed that LPS treatment could promote microglia and the differentiated cells (MLCs) to secret higher level of IL-10 and NO than maDC (Figure 3 d,e). But no difference was detected in the level of TGF-β1, and VEGF (Figure 3d). Further analysis of iNOS confirmed the high expression of NO in MLCs and microglia (Figure 3f).

Therefore, the CNS stromal cells can differentiate maDCs into a kind of cell which has similar morphology, phenotype, phagocytic capacity and cytokine secretion panel with microglia. So we nominated the differentiated cells as microglia-like cells (MLCs).

### MLCs Inhibit maDC-Initiated Proliferation of CD4 T Cells

**Figure 4:**
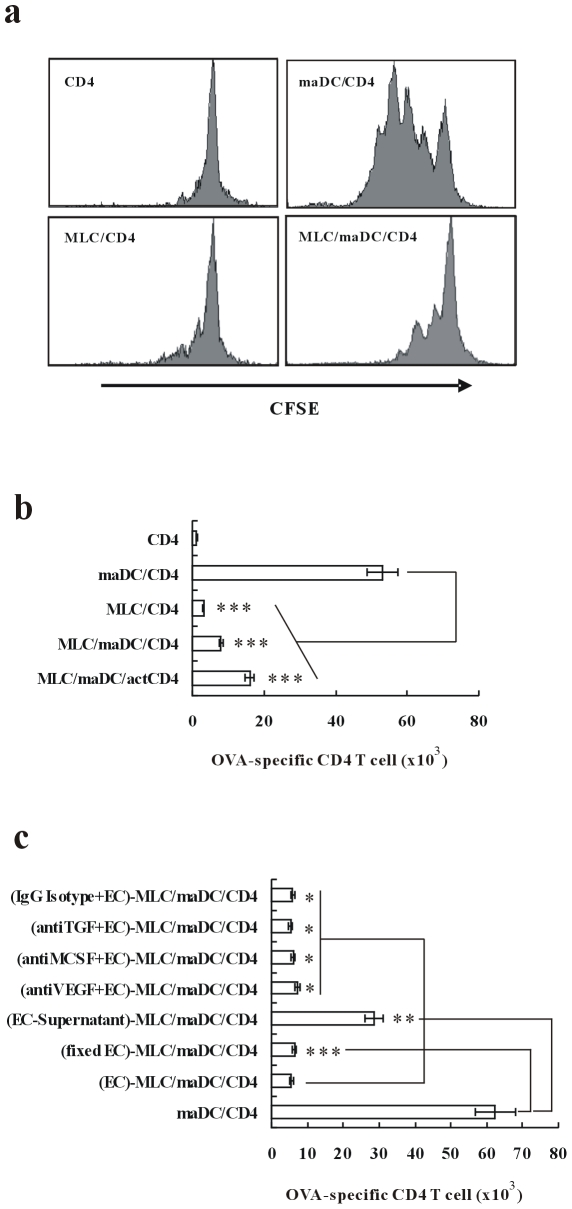
MLCs inhibit maDC-induced proliferation of CD4 T cells. CD4 T cells from DO11.10×C57BL/6 F1 hybrid mice were cocultured with maDCs and/or MLCs for 5 days. (a) Proliferation of CD4 T cells in the coculture system were demonstrated by CFSE dilution. (b) MLCs were added into the maDC/CD4 T coculture system simultaneously or 24 h after maDC coculture with CD4 T cells. Live CD4 T cells in the coculture system were counted by a flow cytometer. Data are presented as mean±s.d. of triplicate wells and representative of three independent experiments. (c) Fixed endothelial cells (EC) or endothelial supernatant were used to culture DC or neutralizing antibodies against TGF-β, M-CSF or VEGF were added into the endothelial-DC coculture system(5 ug/ml, each antibody), then the differentiated MLC were added into the maDC/T cell coculture system. 3 days later, the alive T cells were counted by FACS. ^**^, *P*<0.05. ^***^, *P*<0.01.

Then we tested the immune function of MLCs. Using CFSE-labeled TCR transgenic CD4 T cells, we analyzed the antigen presenting ability of MLCs. The results showed that OVA_323−339_-pulsed MLCs failed to drive the proliferation of naïve TCR transgenic CD4 T cells. Moreover, MLCs could inhibit maDCs-initiated T cell proliferation when they were added into the maDCs/T cells coculture system (Figure.4a). Further study showed that even if the TCR transgenic CD4 T cells have been activated 24 hours before, MLC could exert similar inhibitory function (Figure 4b). Since it has been demonstrated that TGF-β, M-CSF and VEGF are key factors in regulatory DC modulation, to find thier effects on the MLC differentiation, we used fixed endothelial cells (EC) or endothelial supernatant to culture DC or added neutralizing antibodies to the EC-DC coculture system. The results showed that both fixed endothelial cells and endothelial supernatant have effects to promote MLC differentiation, indicating that both cell-to-cell contact and soluble factors played an important role in promoting MLC differentiation. However, blocking any of TGF-β, GM-CSF, M-CSF and VEGF did not abolish the inhibitory function of MLC, indicating that these soluble factors are not the key factors in MLC differentiation, but their roles in the MLC differentiation remained to be investigated.

### NO Is Involved in the Immune Inhibition by Microglia and MLCs

**Figure 5:**
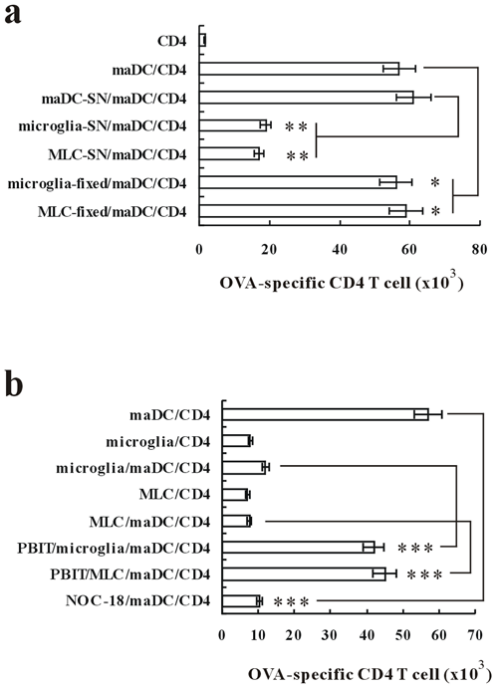
NO is involved in the immune inhibition by microglia and MLCs. (a) The inhibitory function of supernatants(SN) of microglia or MLCs cultured for 48 hours and paraformaldehyde-fixed microglia or MLCs were tested in the mDC/CD4 coculture system. (b) NO donor NOC-18 at a dosage of 40 µg/ml, NO synthetase inhibitor PBIT at a dosage of 20 µg/ml and neutralization antibodies against IL-10 were added into the maDCs/T cells coculture system and then the live CD4 T cells were detected by FACS. Data are presented as mean±s.d. of triplicate wells and representative of three independent experiments.^*^, *P*>0.05;^**^, *P*<0.05; ^***^, *P*<0.01.

To elucidate the factors that lie behind the immune inhibiting function of microglia and MLCs, we examined the inhibiting capability of 4% paraformaldehyde-fixed microglia and MLCs, and the supernatants of microglia and MLCs after culture for 5 days. The results show that 50% supernatant of either microglia or MLCs in the culture system reproduced the immune inhibiting function, while neither of the two kinds of fixed cells had inhibitory function (Figure.5a).

We found both MLCs and microglia had a basic secretion of NO, and there was a burst of NO production upon stimulation with LPS at a dosage of 10 ng/ml or IFN-γ(5 ng/ml). However the NO production of maDCs was very low either in the presence or absence of LPS (Figure 3e). Addition of NO donor NOC-18 at a dosage of 40 µg/ml to the coculture system strongly inhibited the proliferation of CD4 T cells. PBIT, the selective NO synthetase inhibitor, could dramatically abolished the inhibiting effects of microglia and MLCs (Figure 5b). As shown in figure 3d, compared to DC, microglia and microglia-like cell could secret higher level of IL-10, but similar level of TGF-βand VEGF when they are stimulated by LPS. The results indicated that TGF-βand VEGF might not involve in the inhibitory functions of microglia and microglia-like cell. And using neutralizing antibody against IL-10 to block the secreted IL-10 in the microglia (or MLC)/mDC/CD4 coculture system could not abolish the inhibition(data not shown). The data demonstrated that NO played a notable role in the immunological suppressive function of microglia and MLCs.

## Discussion

Microglia originate from bone marrow hematopoietic cells and populate the CNS during early fetal life and remain in the parenchyma of the CNS as resident macrophages. There is much controversy about the correlation of microglia with the CNS immunity. Carson reported that microglia were of incomplete APC phenotype and failed to present peptides to the responder T cells with the defined TCR[10]. We demonstrate further that microglia not only are impotent in presenting peptides to naïve CD4 T cells with defined TCR but also can inhibit antigen activated CD4 T cell expansion. With this immune inhibitory potential, microglia resided in the CNS may contribute to the privilege status of the CNS. Considering the overwhelming numbers of microglia against the paucity of the immune competent DCs and naïve T cells in normal CNS[12], [13], the immune response triggered in the CNS should be strongly pressed by microglia and homeostasis in the CNS should be safely guarded.

Neurodegenerative diseases are often accompanied with neuroinflammatory process in which large numbers of DCs and CD4 T cells are recruited into the CNS and cause irreversible neural impairment[14]. For example, in EAE model, large numbers of activated autoreactive CD4 T cells and DCs were observed in the CNS. The entering of activated myelin specific T cells to CNS is thought to be important for the initiation of EAE[6], [7]. Suter reported that DCs isolated from EAE mice CNS were found to inhibit T cell proliferation stimulated by mature bone marrow-derived maDCs[9]. It is notable that these isolated DCs exhibit a phenotype similar to imDCs characterized by intermediate MHC class II and low CD80 expression, and we presume these inhibitory DCs might be the redifferentiated cells from maDCs under the influence of the CNS microenvironment. We paid attention to this issue in the light of our previous discovery that maDCs in the spleen can be induced by splenic endothelia stroma to differentiate into regulatory DCs which are capable of downregulating immune response by inhibiting T cell proliferation[15]. In this work, we found that maDCs could also be induced by the CNS endothelia to differentiate into inhibitory MLCs which share similar phenotype and phagocytic capability with microglia. It has been reported that GFP positive microglia were found in the brain of the C57BL/6 mice with induced Parkinson's disease after the mice were irradiated and intravenously injected with GFP mice derived bone marrow cells[16]. Taken together, we can conclude that microglia could be replenished by maDCs which have infiltrated into the inflammatory CNS and the CNS stroma might contribute to the redifferentiation of infiltrated maDCs. Differentiation from maDCs to microglia may be one of the fates of maDCs recruited to the CNS in EAE model. This differentiation combined with functional changes from stimulation to inhibition may terminate the immune response in the CNS and result in a remitting course of EAE.

In our study, we found that endothelia from the CNS can differentiate maDCs into microglia-like cells (MLCs), which possess similar phenotype and immune inhibitory function as microglia. Similarly, it is reported that astrocytes secreted factors could drive monocytes or macrophages to differentiate into a microglial which has ramified morphology, overexpressed substance P and the calcium binding protein Iba-1, dimly expressed class II MHC and a potassium inward rectifier current[17]–[20]. Here we focused on the immunological function of microglia and the differentiated cells under the influence of the endothelia stromal cells. All the results indicate that different components of the microenvironment in the CNS might play a key role in different aspects of the infiltrated antigen-presenting cells.

Endothelia is one of the main component of the stromal cells of different organ, which express many kinds of membrane associated extracellular matrix (ECM) molecules, including fibronectin and fibrinogen which are the bioligands of CD11b (integrin αM) and CD11c (integrin αX)[21]. CD11b and CD11c are lineage markers of monocyte-derived immunocytes, including macrophages, microglia, kupffer cells and dendritic cells. In the organs, monocyte-derived cells normally adhere to the endothelia of micrangium and sinusoid through the binding between integrins and their ligands. The adhesion is in favor of functional modulation of the monocyte-derived cells. Endothelia of brain secret high level of TGF-β, M-CSF and VEGF, which have been demonstrated that they are key factors in regulatory DCs modulation[22], [23]. It has been demonstrated that stromal cells (endothelia and fibroblast) can drive the differentiation of mDCs to regulatory DCs[15], [24], [25].

TGF-β, M-CSF and VEGF have been demonstrated to be driven factors for the differentiation of regulatory DC in vitro[25]–[27]. In our manuscript our conclusion is TGF-β,M-CSF and VEGF might not be involved in the inhibitory functions of microglia and MLCs and blocking TGF-β, M-CSF and VEGF can not abolish the differentiation from maDC to MLC. But their effects in the differentiation of MLCs remain to be investigated. Because the EC-DC coculture system is too complex, many soluble factors might combine to promote the differentiation of MLC. No great difference might be observed by blocking a single one but we can not deny its role. Our previous experience showed that no changes were observed by using neutralizing antibody against some cytokine does not mean this cytokine does not play a role. For example, in our previous study, neutralization of TGF-β in the coculture system of splenic stormal cells and mature DC does not alter the differentiation of maDC[15]. But in others' study, it is reported that culture DC with TGF-β could induce differentiation[27]. So the detailed effects of TGF-β, M-CSF and VEGF from endothelia in the CNS on the differentiation of MLC remains to be investigated.

**Figure 6:**
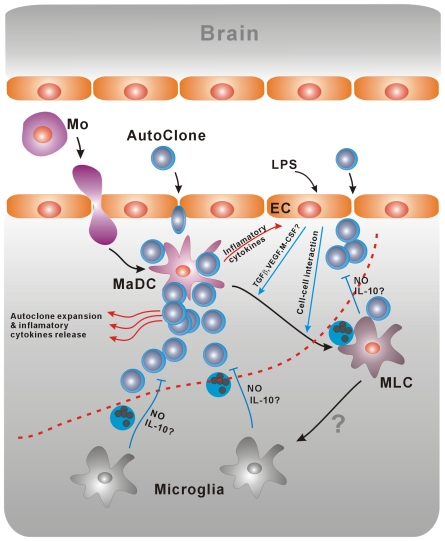
A model of dynamic transformation of antigen presenting cells in CNS. Some special infection in the CNS can destroy neural tissues and endothelial cells of capillary, in this status, BBB was impaired followed by the entrance of monocytes and autoreactive T cells from peripheral blood. Monocytes will become mature DCs in the stimulation of inflammation factors secreted by the infected cells, after crossing the endothelia. New mature DCs derived from monocytes will capture the antigens of neural tissue, prime autoreactive T cells and lead to immunopathological injury. On the contrary, microglia activated by the inflammation factors can secrete high level of NO to inhibit T cell proliferation, even induce T cell apoptosis. The role of IL-10 secreted by microglia in the inhibitory function remains to be investigated. The infiltrated dendritic cells after priming might become inhibitory microglia like cells or microglia under the sustained influence of microenvironment. The cell-to-cell interaction played a key role in the differentiation of DC. The effects of TGF-β, M-CSF, VEGF secreted by activated endothelial cells remain to be investigated. Whether MLC will differentiate into microglia is unknown. Microglia and the transformation from priming dendritic cells to inhibitory microglia like cells and even microglia contribute to the remission of the autoimmune diseases of CNS.

Furthermore, as to the effects of IL-10 in the immunological suppressive function of microglia and MLCs, previous studies have shown that IL-10 play a role in the suppression for immune response[28]. In our system, though MLC secret high level of IL-10, IL-10 is not the key factor of the inhibitory function by blocking antibody. According to the same reasons as above, we can demonstrate IL-10 is not a key factor, but can not deny its effects in the inhibitory functions. As shown in Figure 6, the detailed mechanisms of MLC differentiation and its inhibitory function remain to be investigated.

We rise a hypothesis that in the process of CNS immunopathology, antigen presenting cells (APC) entering the CNS will undergo a functional transformation from priming APC to suppressive APC under the influence of CNS microenvironment. The transformation is dynamic and related with the status of disease. The final transformation to suppressive microglia will suppress the proliferation even induce the apoptosis of autoreactive T cells and terminate the progress of the disease. The microglia and microenvironment of CNS make up the immune barrier of CNS (Figure 6).

Our unpublished data showed that peritoneal macrophages with stimulatory function can be transformed to inhibitory cells by the endothelia stroma of spleen, brain and liver. This indicates that stromal cells have the propensity to induce stimulatory antigen presenting cells to differentiate to inhibitory ones. Combined with our previous study[15], [24], [25], this work might strengthen the hypothesis that the endothelial cells are important in maintaining local immune homeostasis by educating the infiltrated antigen-presenting cells.

## Materials and Methods

### Mice

C57BL/6(H-2K^b^)mice and Balb/c(H-2K^d^) mice were purchased from Vitariver (Beijing, China). OVA_323-339_ peptide specific TCR transgenic mice DO11.10(H-2K^d^) and EGFP transgenic mice C57BL/6-TgN(ACTbEGFP)1Osb(H-2K^b^)were obtained from the Jackson Laboratory(Bar Harbor, ME). All mice were housed and cared according to the approved protocols of the Tsinghua University Animal Care and Use Committee.

### Reagents

7-amino-actinomycin D (7-AAD) and CFSE were purchased from Sigma (St Louis, MO). Magnetic beads-conjugated mAbs to CD4, CD11b, CD11c, CD31, PE were purchased from Miltenyi Biotec (Bergisch Gladbach, Germany). Fluorescein-conjugated mAbs to CD4, CD11b, CD11c, CD40, CD80, CD86, CD31, Ia and isotype control mAbs were purchased from BD Pharmingen (SanDiego, CA).

### Cell Preparation

Newborn C57BL/6 mice brain was scissored into pieces and attached to 24-well plate. After maintained for 2 weeks, the attached cells were digested and incubated with magnetic beads-conjugated mAbs to remove the CD11b positive cells and select the CD11b^−^ CD31^+^ cells which were used as the CNS endothelial stromal cells.

The CNS mononuclear cells were enriched from perfused brain of adult C57BL/6 mice according to the method described[9], [10]. Then the CD11b positive cells were sorted using FACSAria. The purity of the sorted cells is about 95%.

Mature DCs were generated from bone marrow cells in the presence of GM-CSF and IL-4 according to the established protocol [29].

MaDCs derived from C57BL/6 or C57BL/6-TgN(ACTbEGFP)1Osb mice were respectively seeded on the CNS endothelia monolayer (50% confluence) in 24-well plate. After 14 days of coculture, CD11b^+^ cells were purified using magnetic microbeads and used as MLCs.

### RT-PCR Analysis of iNOS Expression

Total RNA was purified from the endothelial stromal cells using an RNAfast200 purification kit (RNAfast200, Fastagen Biotech, Shanghai, China), reverse transcribed and subjected to PCR amplification using the following primers: forward: 5′ ACCACCCTCCTCGTTC 3′, reverse: 5′ GCCTATCCGTCTCGTC 3′.

### Chemotactic Assay

Cell migration was measured in 24-well culture plate with cell culture inserts, First, 600 µl 50% endothelial stromal cell supernatant or control RPMI-1640 medium supplemented with 10%FCS was added into wells in triplicate, then 3 µm pore size insert was placed. The upper chamber of inserts was added with 200 µl monocytes or DC cell suspensions (5×10^4^/ml). After incubation at 37°C in 5% CO_2_ for 6 hours, the insert was removed, and the number of cells in the well was counted by flow cytometry.

### Phagocytic Capability and Phenotype Assay

MLCs, microglia and maDCs were incubated with Alexa488-conjuated OVA and the fluorescence intensity was detected with FACSAria.

For analysis of phenotype, MLCs, microglia and maDCs were blocked with rat serum and 2.4G2 antibody before staining with Fluorescein-conjugated mAbs. And data were acquired with FACSAria.

### Cytokines, Chemokines and NO Measurement

Cytokines and chemokines concentration was assayed by ELISA (ebioscience). NO production was tested by measuring the nitrite concentration with the Griess assay according to the method described[15].

### Assay for Antigen Presenting Function of Microglia and MLCs

According to the method described[15], CD4 T cells from DO11.10×C57BL/6 F1 hybrid mice were obtained by magnetic cell sorting and then cocultured with either maDCs or microglia (or MLCs) for 5 days at a ratio of 1∶10 (DCs/T cells, microglia/T cells, or MLCs/T cells) in 96-well plates (1×10^5^ T cells in 200 µl per well) in the presence of OVA(323−339). Cells were then double stained with anti-CD4-PE and 7-AAD, and the number of CD4^+^ 7-AAD^−^ live cells was counted with FACSAria. For inhibition test, microglia or MLCs were added to the DC/T coculture system in a ratio of 1∶1 (microglia∶DC).

### Statistical Analysis

All experiments were performed at least 3 times. All data analysis was performed using a 2-tailed Student *t* test. *P* value less than 0.05 was considered as statistically significant.
